# Contrasting Associations Between Heart Rate Variability and Brainstem-Limbic Connectivity in Posttraumatic Stress Disorder and Its Dissociative Subtype: A Pilot Study

**DOI:** 10.3389/fnbeh.2022.862192

**Published:** 2022-05-30

**Authors:** Janine Thome, Maria Densmore, Braeden A. Terpou, Jean Théberge, Margaret C. McKinnon, Ruth A. Lanius

**Affiliations:** ^1^Department of Psychiatry, Western University, London, ON, Canada; ^2^Department of Theoretical Neuroscience, Central Institute of Mental Health, Medical Faculty Mannheim, Heidelberg University, Mannheim, Germany; ^3^Department of Psychiatry and Psychotherapy, Central Institute of Mental Health, Medical Faculty Mannheim, Heidelberg University, Mannheim, Germany; ^4^Imaging Division, Lawson Health Research Institute, London, ON, Canada; ^5^Homewood Research Institute, Guelph, ON, Canada; ^6^Department of Psychiatry and Behavioural Neurosciences, McMaster University, Hamilton, ON, Canada; ^7^Department of Medical Biophysics, Western University, London, ON, Canada; ^8^Mood Disorders Programs, St. Joseph’s Healthcare Hamilton, Hamilton, ON, Canada; ^9^Department of Neuroscience, Schulich School of Medicine & Dentistry, University of Western Ontario, London, ON, Canada

**Keywords:** posttraumatic stress disorder, resting state functional magnetic resonance imaging, heart rate variability, brainstem, reticular activating system

## Abstract

**Background:**

Increasing evidence points toward the need to extend the neurobiological conceptualization of posttraumatic stress disorder (PTSD) to include evolutionarily conserved neurocircuitries centered on the brainstem and the midbrain. The reticular activating system (RAS) helps to shape the arousal state of the brain, acting as a bridge between brain and body. To modulate arousal, the RAS is closely tied to the autonomic nervous system (ANS). Individuals with PTSD often reveal altered arousal patterns, ranging from hyper- to blunted arousal states, as well as altered functional connectivity profiles of key arousal-related brain structures that receive direct projections from the RAS. Accordingly, the present study aims to explore resting state functional connectivity of the RAS and its interaction with the ANS in participants with PTSD and its dissociative subtype.

**Methods:**

Individuals with PTSD (*n* = 57), its dissociative subtype (PTSD + DS, *n* = 32) and healthy controls (*n* = 40) underwent a 6-min resting functional magnetic resonance imaging and pulse data recording. Resting state functional connectivity (rsFC) of a central node of the RAS – the pedunculopontine nuclei (PPN) – was investigated along with its relation to ANS functioning as indexed by heart rate variability (HRV). HRV is a prominent marker indexing the flexibility of an organism to react adaptively to environmental needs, with higher HRV representing greater effective adaptation.

**Results:**

Both PTSD and PTSD + DS demonstrated reduced HRV as compared to controls. HRV measures were then correlated with rsFC of the PPN. Critically, participants with PTSD and participants with PTSD + DS displayed inverse correlations between HRV and rsFC between the PPN and key limbic structures, including the amygdala. Whereas participants with PTSD displayed a *positive* relationship between HRV and PPN rsFC with the amygdala, participants with PTSD + DS demonstrated a *negative* relationship between HRV and PPN rsFC with the amygdala.

**Conclusion:**

The present exploratory investigation reveals contrasting patterns of arousal-related circuitry among participants with PTSD and PTSD + DS, providing a neurobiological lens to interpret hyper- and more blunted arousal states in PTSD and PTSD + DS, respectively.

## Introduction

Arousal is critical to the conceptualization of posttraumatic stress disorder (PTSD) since it is a dynamic disorder that includes arousal states ranging from hyperarousal (e.g., hypervigilance, altered startle threshold) to more blunted arousal patterns associated with emotional detachment, depersonalization, and derealization. These latter states are more frequently associated with the dissociative subtype of PTSD (PTSD + DS) (Wolf et al., 2012a,b; APA, 2013; Seligowski et al., 2019; for review see Hansen et al., 2017; Fenster et al., 2018; Lanius et al., 2018; van Huijstee and Vermetten, 2018).

On a neural level, hyperarousal states have been found to be associated with reduced activation of brain regions underlying cognitive control (e.g., ventromedial prefrontal cortex) and enhanced activation of brain regions underlying emotion generation (e.g., amygdala, periaqueductal gray), while the reverse pattern has been implicated in more blunted arousal states (e.g., Fenster et al., 2018; Lanius et al., 2018; see also Lebois et al., 2021). Critically, the aforementioned interaction profile between subcortical and cortical brain regions has recently been extended to include deep-layer brain regions involved in arousal and innate reflexive function (Harricharan et al., 2016; see also Holmes et al., 2018; Olive et al., 2018; Rabellino et al., 2018a,^2019^; Brandão and Lovick, 2019; Terpou et al., 2019b,^2020^, ^2022^; Thome et al., 2019; Haubrich et al., 2020; Lanius et al., 2020; Webb et al., 2020). Specifically, as compared to healthy controls and PTSD, individuals with PTSD + DS show increased resting state functional connectivity of the pedunculopontine nuclei (PPN) – a key node of the RAS – with the ventromedial prefrontal and anterior cingulate cortices, as well as with limbic regions, including the amygdala and parahippocampal gyrus (Thome et al., 2019). These findings highlight alterations in deeper-layer neuronal circuitries critical to promoting and modulating arousal in individuals with PTSD and its dissociative subtype.

The autonomic nervous system (ANS) plays an important role in coordinating and generating arousal, which is often measured via peripheral changes, such as heart rate, blood pressure, and skin conductance (Berntson et al., 1991; Cacioppo et al., 2007; Mendes, 2009). This is achieved by a flexible recruitment of the two branches of the ANS: the sympathetic (SNS) and the parasympathetic nervous system (PNS). The activity of the heart can be used as an indicator of the interaction between the SNS and the PNS, reflecting sympathoexcitation (i.e., tachycardia) and sympathoinhibition states (i.e., bradycardia), respectively (Koepchen, 1984; Sinski et al., 2006; Coote and Chauhan, 2016; Laborde et al., 2017). This variable interaction generates changes in the inter-beat intervals of the heart rate over time (i.e., heart rate variability, HRV), where higher HRV has been related to effective adaptation to the environment (see e.g., Thayer et al., 2012). To estimate the influence of sympathetic and parasympathetic contributions on HRV, different information can be extracted from the cardiac signal. Whereas fast frequencies of the cardiac signal are more likely to reflect parasympathetic activation (e.g., high frequency HRV, HF-HRV), slow frequencies are more likely to be associated with sympathetic activation (e.g., low frequency HRV, LF-HRV) (Malik et al., 1996). It is important to note, however, that this distinction is not so clear-cut (Billman, 2013; Shaffer and Ginsberg, 2017). In particular, the LF component seems to be influenced by a mixture of SNS and PNS activation (Randall et al., 1991; Ahmed et al., 1994; Billman, 2009, 2011), a potential limitation to be considered moving forward. The ANS is tightly linked to the central autonomic network (CAN), where a bidirectional communication between these two systems is critical to the connection between brain and body (e.g., Thayer et al., 2012; Beissner et al., 2013; Chang et al., 2013; Mather and Thayer, 2018; Riganello et al., 2019). The CAN comprises brainstem (e.g., nucleus tractus solitarius, locus coeruleus), midbrain (e.g., periaqueductal gray, superior colliculi), as well as subcortical (e.g., thalamus, insula, amygdala) and higher cortical brain regions (e.g., anterior and midcingulate cortex, ventromedial prefrontal cortex, posterior cingulate cortex), where brainstem structures are thought to generate the output to the heart (e.g., Beissner et al., 2013; Valenza et al., 2018). The complex interplay between the ANS and the CAN enables autonomic flexibility, prefrontal-directed control, and the capacity to re-establish homeostasis after a stressful event (Beissner et al., 2013; Mather and Thayer, 2018).

Critically, PTSD has been associated with alterations in ANS-modulated responses (see also Shalev et al., 1998; Videlock et al., 2008; APA, 2013; Depierro et al., 2013; Lang et al., 2016, 2018; Rabellino et al., 2017; Niles et al., 2018; Herzog et al., 2019; Lis et al., 2019). In particular, individuals with PTSD are generally characterized by a reduced parasympathetic-related HRV at rest (Hauschildt et al., 2011; Kamkwalala et al., 2012; Dennis et al., 2016b; Rissling et al., 2016; for a meta-analyses see Campbell et al., 2019; Spiller et al., 2019; Schneider and Schwerdtfeger, 2020). Studies examining sympathetic-related HRV in PTSD are mixed, with most studies reporting reduced HRV (Chang H.-A. et al., 2013; Shah et al., 2013; Wahbeh and Oken, 2013; Dennis et al., 2014, 2016a; Rissling et al., 2016), while others have reported enhanced HRV (Cohen et al., 1998, 2000; Lakusic et al., 2007; for meta-analyses see Nagpal et al., 2013; Chalmers et al., 2014; Schneider and Schwerdtfeger, 2020; see also Siciliano et al., 2022). Importantly, these contrasting findings may be driven partially by dissociative symptomatology. Several studies examining the relationship between dissociation and HRV in different populations have reported enhanced recruitment of the PNS in response to threatful or trauma-related cues, thus pointing toward a relationship between dissociation and sympathoinhibitory responses (Farina et al., 2015; see also Owens et al., 2015; Chou et al., 2018; Schäflein et al., 2018; Herzog et al., 2019; Krause-Utz et al., 2019).

Despite the significant amount of evidence pointing to HRV and arousal-related neural alterations in PTSD, research investigating the relationship between HRV and functional brain responses is still in its nascent stages. Previous findings by Rabellino et al. (2017) have reported altered recruitment of CAN-related brain regions in association with parasympathetic-related HRV in participants with PTSD. In particular, whereas brain regions related to the processing of bodily signals (i.e., anterior and posterior insula) showed reduced recruitment during trauma-related stimulus processing, brain regions underlying emotion processing (i.e., amygdala) displayed enhanced recruitment in participants with PTSD as compared to healthy controls (Rabellino et al., 2017). Similar findings have also been reported at rest (Thome et al., 2017). Furthermore, Grupe et al. (2020) investigated neural responses of the anticipation of predictable and unpredictable conditioned threat and safety cues in individuals with high and low PTSD symptoms related to combat. A relationship between HRV and higher-order cognitive control systems was observed during unpredictable threat vs. safety trials. Moreover, reduced activation of the ventromedial prefrontal cortex was related to lower parasympathetic-related HRV in individuals with higher PTSD symptoms during the anticipation of safety trials.

To date, however, there are no studies examining the relationship between deep-layer neurocircuitries and autonomic responses in PTSD and its dissociative subtype. This is of particular importance given that rodent studies have revealed that the RAS, specifically the PPN, projects to the amygdala, presenting a direct pathway for arousal (Pahapill and Lozano, 2000; Alam et al., 2011). Accordingly, the present investigation aims to examine the exploratory relationship between resting state functional connectivity of the pedunculopontine nuclei (PPN) with resting sympathetic- and parasympathetic-related HRV in individuals with PTSD, its dissociative subtype (PTSD + DS), and controls.

We hypothesized reduced parasympathetic- and reduced sympathetic-related HRV in PTSD and its dissociative subtype, respectively, as compared to controls. Individuals with PTSD were hypothesized to show a altered relationship between parasympathetic-related HRV and RAS functional connectivity to emotion processing brain regions of the CAN (i.e., limbic structures, midbrain) as compared to controls and PTSD + DS. Individuals with PTSD + DS were hypothesized to exhibit a altered relationship between sympathetic-related HRV and RAS functional connectivity to regulatory brain regions of the CAN (i.e., ventromedial prefrontal and anterior cingulate cortices) as compared to controls and PTSD.

## Materials and Methods

### Sample Description

The present investigation included 130 participants, of whom 89 participants met the criteria for PTSD, while 40 participants were free of any mental disorder throughout their life (control group). Of the 89 individuals who met criteria for PTSD, 57 individuals met criteria for PTSD, and 32 met criteria for the dissociative subtype of PTSD (PTSD + DS). Here, we included a sub-sample of a previously published larger sample, as we excluded participants with non-exploitable pulse data (Harricharan et al., 2016, 2017; Nicholson et al., 2016, 2018; Thome et al., 2017, 2019; Rabellino et al., 2018b,c; Terpou et al., 2018). Scanning took place either at the Robarts Research Institute’s Centre for Functional and Metabolic Mapping or the Lawson Health Research Institute for Imaging in London, ON, Canada. This retrospective study was approved by the Research Ethics Board at Western University.

Posttraumatic stress disorder diagnoses and symptom severity were assessed using the Clinician-Administered PTSD Scale (CAPS IV, CAPS 5; Blake et al., 1995), while comorbid Axis I disorders were diagnosed with the Structured Clinical Interview for DSM-IV Axis I Disorders (SKID-I; First, 1997) by a trained clinical psychologist. For information on additional psychometric measurements, statistical analyses and exclusion criteria please see Table 1 and Supplementary Material 1.

**TABLE 1:** Demographic and clinical characteristics of the study sample.

						*Post hoc* comparison									
	
	controls	PTSD	PTSD + DS			Controls vs. PTSD			Controls vs. PTSD + DS				PTSD vs. PTSD + DS		
	*N* = 40	*N* = 57	*N* = 32	Test-statistic	*p*-value	Test-statistic	df	*p*-value	Test-statistic	df	*p*-value		Test-statistic	df	*p*-value
**Demographics**															
Gender (n)				4.72	0.095										
Female	26	31	6			–	–	–	–	–	–		–	–	–
Male	14	24	24			–	–	–	–	–	–		–	–	–
age mean (SD)	35.28 (11.53)	39.21 (11.94)	39.26 (14.19)	1.39	0.251	–	–	–	–	–	–		–	–	–
**Clinical characteristics**															
CAPS total mean (SD)	0.80 (3.06)	56.04 (20.18)	69.35 (20.36)	171.25	<0.001**	17.14	95	<0.001**	20.99	58	<0.001	**	2.53	75	0.013**
BDI mean (SD)	1.18 (2.14)	23.12 (8.71)	35.60 (11.75)	154.20	<0.001**	15.16	86	<0.001**	17.71	66	<0.001	**	5.43	78	<0.001**
MDI				236.000	<0.001**										
MDI total mean (SD)	34.26 (4.29)	53.81 (13.49)	76.69 (19.82)			8.72	90	<0.001**	12.99	66	<0.001	**	6.19	80	<0.001**
MDI derealization mean (SD)	5.28 (0.69)	8.49 (2.89)	12.24 (3.87)			6.76	90	<0.001**	11.03	66	<0.001	**	4.96	80	<0.001**
MDI depersonalization mean (SD)	5.21 (0.69)	6.77 (2.66)	11.62 (4.99)			3.59	90	<0.001**	7.95	66	<0.001	**	5.75	80	<0.001**
**Trauma history**															
CTQ															
CTQ total mean (SD)	32.37 (9.67)	56.92 (22.89)	65.00 (19.85)			6.21	88	<0.001*	8.86	65	<0.001	**	1.59	79	0.115
Emotional abuse mean (SD)															
Physical abuse mean (SD)															
Sexual abuse mean (SD)															
Emotional neglect mean (SD)															
Physical neglect mean (SD)															
Combat exposure															
Yes (N)															
No (N)															
**State characteristics**															
RSDI dissociation mean (SD)	2.66 (0.38)	3.82 (1.48)	4.92 (1.88)	19.24	<0.001**	4.55	81	<0.001**	6.92	52	<0.001	**	2.53	65	0.014**
STAI-S mean (SD)	3.26 (0.56)	6.00 (2.37)	5.94 (2.22)	23.29	<0.001**	6.70	81	<0.001**	6.82	52	<0.001	**	0.08	65	0.93
**Cardiac response**															
HF-HRV mean (SD)	6.35 (1.08)	5.69 (1.22)	5.85 (1.17)	3.82	0.024**	2.72	95	0.008**	1.87	70	0.065		0.59	87	0.554
LF-HRV mean (SD)	1.86 (0.13)	1.72 (0.23)	1.70 (0.22)	6.72	0.002**	3.25	95	0.002**	3.51	70	0.001	**	0.40	87	0.688
RMSSD mean (SD)	3.72 (0.49)	3.43 (0.54)	3.49 (0.56)	3.67	0.028**	2.66	95	0.009**	1.99	70	0.050	*	0.31	87	0.768

**p < 0.05 (post hoc tests: Bonferroni-adjustment), *p < 0.05 (post hoc tests: without Bonferroni-adjustment), °F-Test, ∧Kruskal-Wallis Test, ^T^T-Test. CAPS, Clinician Administered PTSD Scale; CTQ, Childhood Trauma Questionnaire; HF-HRV, high frequency heart rate variability; LF-HRV, low frequency heart rate variability; MDI, Multiscale Dissociation Inventory; RMSSD, root-mean square of successive differences; RSDI, Response to Script Driven Imagery Scale; STAI-S, State-Trait Anxiety Inventory-State part; PTSD, posttraumatic stress disorder; PTSD + DS, PTSD with dissociative subtype.

### Cardiac Signal Processing

Pulse data were recorded using a finger-tip pulse oximeter (Powerlab 8/35, LabChart 7 Pro) during the 6-min resting functional MRI scanning procedure and sampled at 200 Hz (Robarts Research Institute) or 40 Hz (Lawson Health Science Institute). Pulse data acquired at 40 Hz were re-sampled at 200 Hz by linear interpolation. Peak detection and visual inspection (artifact detection) of the pulse signal was ensured via self-written Matlab scripts (Matlab R2019b; MathWorks). The inspected inter-beat interval (IBI) time series were imported to the KUBIOS Heart Rate Variability Software Package (Kubios Standard; Kuopio, Finland; version 3.2.0) (Tarvainen et al., 2014). IBI data were re-sampled at 4 Hz and HRV measurements were computed, namely a time domain estimate (i.e., root-mean square of successive differences, RMSSD, parasympathetic-related HRV), two frequency domain estimates, high-frequency HRV (HF-HRV, parasympathetic-related HRV) and low frequency HRV (LF-HRV, sympathetic-related HRV) following the task force of the european society of cardiology and the north american society of pacing and electrophysiology guidelines (1996). To calculate HF-HRV (bandpass: 0.15–0.4 Hz) and LF-HRV (bandpass: 0.04–0.15 Hz), spectral analysis using a Fast Fourier transformation based on the Welch’s periodogram method was computed, with the latter employing a window width of 300 s and a window overlap of 50% (Tarvainen et al., 2014). To obtain better approximations of normal distributions of RMSSD, HF-HRV and LF-HRV, metrics were transformed by natural logarithm.

Group differences in HRV were assessed with multivariate analyses of variance. Analyses were performed using SPSS (version 25; SPSS Inc., United States).

### Resting State fMRI Data Acquisition

Functional magnetic resonance imaging (fMRI) was conducted using a 3.0 T whole-body MRI scanner (Magnetom Tim Trio, Siemens Medical Solutions, Erlangen, Germany) with a manufacturer’s 32-channel phased array head coil. T1-weighted anatomical images were collected with 1 mm isotropic resolution (MP-RAGE, TR/TE/TI = 2,300 ms/2.98 ms/900 ms, FA 9°, FOV = 256 mm × 240 mm × 192 mm, acceleration factor = 4, total acquisition time = 192 s). Blood-oxygenation-level dependent (BOLD) fMRI images were obtained with the standard gradient echo planar imaging (EPI) pulse sequence. EPI volumes were acquired with 2 mm isotropic resolution (FOV = 192 mm × 192 mm × 128 mm (94 × 94 matrix, 64 slices), TR/TE = 3,000 ms/20 ms, flip angle = 90°, 120 volumes).

Participants were instructed to close their eyes and let their minds wander during the 6-min resting scan in accordance with standard methods (Bluhm et al., 2011; Harricharan et al., 2016).

### fMRI Data Preprocessing

Image preprocessing and statistical analyses of the fMRI data were conducted using Statistical Parametric Mapping (SPM 12, Wellcome Trust Center of Neuroimaging, London, United Kingdom^1^) and the spatially unbiased infratentorial template (SUIT) toolbox (Version, 3.1; Diedrichsen, 2006; Diedrichsen et al., 2011) implemented in Matlab R2019b (MathWorks).

Functional images were realigned to the first image and then re-sliced to the mean functional image. Six realignment parameters for changes in motion across the different planes were derived. Motion correction was ensured by computing regressors accounting for motion outliers with the Artifact Detection Tool (ART) software package (2 mm motion threshold; ART software; Gabrieli Lab; McGovern Institute for Brain Research, Cambridge, MA, United States^2^; see also Power et al., 2012, 2014), which were incorporated in the analyses in addition to the six movement regressors computed during standard realignment.

#### fMRI Data Preprocessing: Brainstem and Cerebellum

Functional data were normalized to the spatially unbiased infratentorial template (SUIT, version 3.1.; Diedrichsen, 2006; Diedrichsen et al., 2011) toolbox to improve the resolution of the brainstem and the cerebellum across participants, which, in turn, enhanced the signal extracted from the PPN subject-specifically. For details see Supplementary Material 1 (Terpou et al., 2018, 2019a; Thome et al., 2019).

#### fMRI Data Preprocessing: Whole Brain

The realigned and resliced functional images (see section “fMRI Data Preprocessing”) were co-registered to the anatomical image for each subject. Co-registration was followed by the segmentation of the images into each tissue type (gray and white matter as well as cerebrospinal fluid), spatial normalization to the MNI standard template, smoothing with a 6 mm FWHM Gaussian kernel, and band-pass filtering with a high-pass filter of 0.01 Hz and low-pass filter of 0.08 Hz (Bär et al., 2016; Wagner et al., 2018).

### Resting State Functional Connectivity Analyses (rsFC)

#### Seed Region Definition

Seed masks for the right and the left PPN were generated using the WFU PickAtlas (Functional MRI Laboratory, Wake Forest University School of Medicine; Maldjian et al., 2003) by defining 4 mm spheres around the following coordinates: *x* = ± 7, *y* = −32, *z* = −22 (Fling et al., 2013; see also Thome et al., 2019), and confirmed visually using the Duvernoy’s Atlas (Naidlich, 2009). The mean signal BOLD time course of each seed (i.e., the right and the left PPN) was extracted from the data normalized to the SUIT template, ensuring enhanced spatial accuracy of the defined seed regions (self-written MATLAB scripts).

#### First Level

For each seed, separate voxel-wise first-level multiple regression models were generated with the seed time course included as a regressor of interest, while ART-computed regressors (i.e., motion outliers) and realignment parameters were included as regressors of no interest.

#### Second Level: Within-Group Analyses

To explore the interaction of HRV with the rsFC patterns of the PPN within groups, separate multiple regression models (i.e., right PPN, left PPN) were conducted, where each HRV estimate was included as a regressor (i.e., RMSSD, HF-HRV, LF-HRV). Positive as well as negative relationships between PPN rsFC and HRV were investigated. These findings are reported in the Supplementary Material 2 (see Supplementary Tables 1–6).

#### Second Level: Between-Group Analyses

To compare the relationship between the rsFC patterns of the PPN and HRV between groups, we utilized a flexible factorial design with the factor group (controls vs. PTSD vs. PTSD + DS), the factor hemisphere (left PPN vs. right PPN), and interaction terms of “group × hemisphere”, and “group × HRV” covariate for each HRV estimate included (i.e., RMSSD, HF-HRV, LF-HRV). If necessary, *Post hoc* T-contrasts with respect to the interaction term “group × HRV” covariates were conducted to compare each group to one another (i.e., controls vs. PTSD, controls vs. PTSD + DS, PTSD vs. PTSD + DS).

#### Second Level: Direction Relationship of rsFC and HRV

To investigate identified group differences in PPN rsFC patterns and its relation to HRV, contrast estimates of the identified peak coordinates were extracted and correlated with HRV estimates.

#### Analyses Approach and Statistical Thresholding

Resting state functional connectivity was analyzed using a region-of-interest (ROI) approach, with *a priori* brain regions, including the amygdala, the insula, and the ventromedial prefrontal cortex, which were selected due to their relation to the central autonomic network (CAN) (Thayer et al., 2012; Beissner et al., 2013). Bilateral amygdalae, insulae, and ventromedial prefrontal masks were adopted from the automated anatomical labeling atlas (Tzourio-Mazoyer et al., 2002), which was implemented in the WFU PickAtlas (Maldjian et al., 2003).

All ROI results were reported at a local significance threshold of *p* < 0.05 (voxel-level), with an alpha-adjustment for multiple comparisons (family-wise error (FWE) correction). In addition, a Bonferroni adjustment was applied according to the number of tested ROIs (*N* = 3), leading to a local significance threshold of *p* < 0.017, FWE corrected.

## Results

### Sample Characteristics

Groups did not differ in age or gender (see Table 1). Significant group differences emerged for all clinical and subjective experience measurements. Please see Table 1 for details.

### HRV Characteristics

#### HF-HRV

Groups differed in HF-HRV (*F* = 3.82, *p* = 0.024). Control subjects exhibited higher HF-HRV as compared to individuals with PTSD (*T*_95_ = 2.72, *p* = 0.008). No differences were observed between controls and individuals with PTSD + DS (*T*_70_ = 1.87, *p* = 0.065), nor between individuals with PTSD and individuals with PTSD + DS (*T*_87_ = 0.59, *p* = 0.554) (Table 1).

#### LF-HRV

Groups differed in LF-HRV (*F* = 6.72, *p* = 0.002). Control subjects exhibited higher LF-HRV as compared to individuals with PTSD (*T*_95_ = 3.25, *p* = 0.002) and PTSD + DS (*T*_70_ = 3.51, *p* = 0.001). No differences were observed between PTSD and PTSD + DS groups (*T*_87_ = 0.40, *p* = 0.688) (Table 1).

#### RMSSD

Groups differed in RMSSD (*F* = 3.82, *p* = 0.028). Control subjects exhibited increased RMSSD as compared to individuals with PTSD (*T*_95_ = 2.66, *p* = 0.009) and trended toward increased RMSSD when compared to individuals with PTSD + DS (*T*_70_ = 1.99, *p* = 0.050). No differences were observed between PTSD and PTSD + DS groups (*T*_87_ = 0.31, *p* = 0.768) (Table 1).

### Group Differences in PPN Resting State Functional Connectivity Associated With HRV

#### PPN rsFC and HF-HRV

##### PPN rsFC and HF-HRV: Flexible Factorial

The flexible-factorial ANOVA showed a main effect of group (*p*_*FWE*_ = 0.025; please note alpha-level adjustment with FWE correction, without additional Bonferroni correction). Groups differed in rsFC of the PPN with a cluster encompassing the left anterior cingulate cortex and the ventromedial prefrontal cortex (*p*_*FWE*_ = 0.025). Moreover, a significant interaction of “group × HF-HRV” emerged (*p*_*FWE*_ = 0.007). Groups differed in rsFC of the PPN associated with RMSSD with a cluster encompassing the amygdala and the parahippocampal gyrus (*p*_*FWE*_ = 0.007). See Table 2.

**TABLE 2:** ROI-related between-group comparisons of resting state functional connectivity of the left and right pedunculopontine nuclei in association with HF-HRV.

								Peak MNI coordinate		
	L/R	Brain region		k	Z	*p*_*FWE–corr*_	*p*_*uncorr*_	x	y	z
**Main effect of group**										
	L	Ventromedial prefrontal cortex/anterior cingulate cortex	*	411	3.60	0.025	<0.001	−2	50	−12
**Interaction group × HF-HRV**										
	R	Amygdala/parahippocampal gyrus	**	85	3.65	0.007	<0.001	30	−4	−14
***Post Hoc*: interaction group × HF-HRV**										
Controls > PTSD			n.s.							
Controls > PTSD + DS	R	Amygdala/parahippocampal gyrus	**	68	3.43	0.012	<0.001	32	−4	−14
PTSD > controls			n.s.							
PTSD > PTSD + DS	R	Amygdala/parahippocampal gyrus	**	80	4.09	0.001	<0.001	30	−4	−14
PTSD + DS > controls			n.s.							
PTSD + DS > PTSD			n.s.							

Region of interest (ROI) approach: RsFC results are reported at a local significance threshold of *p < 0.05, and **p < 0.017 (i.e., Bonferroni-adjustment according to the number of applied ROIs), FWE corrected. HF-HRV, high-frequency heart rate variability; PTSD, posttraumatic stress disorder; PTSD + DS, PTSD with the dissociative subtype; L, Left; R, Right; n.s., no significant difference; k, cluster size; p_uncorr_, p-value, uncorrected for multiple comparisons; p_FWEcorr_, p-value, corrected for multiple comparisons (family-wise error).

##### PPN rsFC and HF-HRV: Controls vs. PTSD

We did not observe significant differences in rsFC of the PPN associated with HF-HRV with any predefined ROI when comparing controls to individuals with PTSD (i.e., controls > PTSD or PTSD > controls).

##### PPN rsFC and HF-HRV: Controls vs. PTSD + DS

**FIGURE 1:**
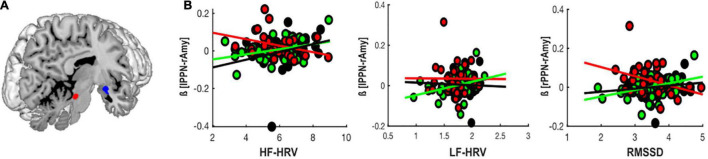
Group-differences in PPN rsFC with CAN-related brain regions associated with HRV. **(A)** Group-differences in PPN (see region highlighted in red) rsFC with limbic brain regions (i.e., the right amygdala and the parahippocampal gyrus in blue) associated with HF-HRV and RMSSD. **(B)** Correlation of beta-values extracted from the peak voxel of the identified group difference in PPN rsFC with limbic brain regions and HF-HRV, LF-HRV, and RMSSD in controls (black), PTSD (green), and PTSD + DS (red), respectively. Amy, amygdala; HF-HRV, high-frequency heart rate variability; LF-HRV, low-frequency heart rate variability; lPPN, left peduncolopontine nuclei; RMSSD, root-mean square of successive differences.

As compared to individuals with PTSD + DS, controls showed significantly stronger rsFC of the PPN associated with HF-HRV with a cluster encompassing the amygdala and the parahippocampal gyrus (*p*_*FWE*_ = 0.012). See Table 2 and Figure 1A.

As compared to controls, PTSD + DS did not show any significant differences in rsFC of the PPN associated with HF-HRV with any predefined ROI.

##### PPN rsFC and HF-HRV: PTSD vs. PTSD + DS

As compared to individuals with PTSD + DS, individuals with PTSD showed significantly stronger rsFC of the PPN associated with HF-HRV with a cluster encompassing the amygdala and the parahippocampal gyrus (*p*_*FWE*_ = 0.001). See Table 2 and Figure 1A.

As compared to PTSD, PTSD + DS did not show any significant differences in rsFC of the PPN associated with HF-HRV with any predefined ROI.

#### PPN rsFC and LF-HRV

##### PPN rsFC and LF-HRV: Flexible Factorial

The flexible-factorial ANOVA did not reveal a main effect of group, a “group × hemisphere,” nor a “group × LF-HRV” interaction. Moreover, exploratory *post hoc* contrasts did not reveal differences between the rsFC of the PPN and any other brain region associated with LF-HRV between groups.

#### PPN rsFC and RMSSD

##### PPN rsFC and RMSSD: Flexible Factorial

The flexible-factorial ANOVA showed a main effect of group (*p*_*FWE*_ = 0.036; please note alpha-level adjustment with FWE correction, without additional Bonferroni correction). Groups differed in rsFC of the PPN with a cluster encompassing the left anterior cingulate cortex and the ventromedial prefrontal cortex (*p*_*FWE*_ = 0.036).

Moreover, a significant interaction of “group × HF-HRV” emerged (*p*_*FWE*_ = 0.024; please note alpha-level adjustment with FWE correction, without additional Bonferroni correction). Groups differed in rsFC of the PPN associated with RMSSD with a cluster encompassing the amygdala and the parahippocampal gyrus (*p*_*FWE*_ = 0.024). See Table 3.

**TABLE 3:** ROI-related between-group comparisons of resting state functional connectivity of the left and right pedunculopontine nuclei in association with RMSSD.

								Peak MNI coordinate		
	L/R	Brain region		k	Z	*p*_*FWE–corr*_	*p*_*uncorr*_	x	y	z
**Main effect of group**										
	L	Ventromedial prefrontal cortex/anterior cingulate cortex	*	391	3.48	0.036	<0.001	−2	50	−12
**Interaction croup × HF-HRV**										
	R	Amygdala/parahippocampal gyrus	*	74	3.25	0.024	<0.001	30	−4	−14
***Post Hoc*: interaction group × HF-HRV**										
Controls > PTSD			n.s.							
Controls > PTSD + DS	R	Amygdala/parahippocampal gyrus	*	67	2.97	0.045	<0.001	32	−4	−16
PTSD > controls			n.s.							
PTSD > PTSD + DS	R	Amygdala/parahippocampal gyrus	**	75	3.81	0.003	<0.001	30	−4	−14
PTSD + DS > controls			n.s.							
PTSD + DS > PTSD			n.s.							

Region of interest (ROI) approach: RsFC results are reported at a local significance threshold of *p < 0.05, and **p < 0.017 (i.e., Bonferroni-adjustment according to the number of applied ROIs), FWE corrected. RMSSD, root-mean square of successive differences; PTSD, posttraumatic stress disorder; PTSD + DS, PTSD with the dissociative subtype; L, Left; R, Right; n.s., no significant difference; k, cluster size; p_uncorr_, p-value, uncorrected for multiple comparisons; p_FWEcorr_, p-value, corrected for multiple comparisons (family-wise error).

##### PPN rsFC and RMSSD: Controls vs. PTSD

We did not observe any significant differences in rsFC of the PPN associated with RMSSD with any predefined region of interest when comparing controls to PTSD (i.e., controls > PTSD; PTSD > controls).

##### PPN rsFC and RMSSD: Controls vs. PTSD + DS

As compared to individuals with PTSD + DS, controls showed significantly stronger rsFC of the PPN associated with RMSSD with a cluster encompassing the amygdala and the parahippocampal gyrus (*p*_*FWE*_ = 0.045; please note alpha-level adjustment with FWE correction, without additional Bonferroni correction). See Table 3.

As compared to controls, PTSD + DS did not demonstrate significant differences in rsFC of the PPN associated with RMSSD with any predefined region of interest.

##### PPN rsFC and RMSSD: PTSD vs. PTSD + DS

As compared to individuals with PTSD + DS, PTSD showed a significantly stronger rsFC of the PPN associated with RMSSD with a cluster encompassing the amygdala and the parahippocampal gyrus (*p*_*FWE*_ = 0.003). See Table 3.

As compared to PTSD, PTSD + DS did not show any significant differences in rsFC of the PPN associated with RMSSD with any predefined region of interest.

### Direction of PPN Resting State Functional Connectivity and HRV

In controls and individuals with PTSD, lower HRV was related to a reduced connectivity between the PPN and the right amygdala (LPPN: HF-HRV controls: *r* = 0.24, *p* = 0.025; RPPN: LF-HRV PTSD: *r* = 0.22, *p* = 0.015; RPPN: RMSSD PTSD: *r* = 0.21, *p* = 0.021). By contrast, in PTSD + DS, lower HRV was related to an enhanced connectivity between the PPN and the right amygdala (LPPN: HF-HRV *r* = −0.3, *p* = 0.013). See Figure 1B.

## Discussion

The present exploratory investigation examined resting state functional connectivity (rsFC) of the reticular activating system (RAS), specifically the pedunculopontine nuclei (PPN), and its association with heart rate variability (HRV) in individuals with PTSD, its dissociative subtype (PTSD + DS), and healthy controls. Whereas individuals with PTSD and PTSD + DS revealed reduced LF-HRV, only individuals with PTSD showed reduced HF-HRV as compared to controls. No differences in HRV were observed between PTSD and PTSD + DS. Importantly, however, individuals with PTSD and PTSD + DS displayed inverse correlations between HRV and rsFC between the PPN and key limbic structures, including the amygdala. Whereas participants with PTSD displayed a *positive* relationship between HRV and PPN rsFC with the amygdala, participants with PTSD + DS displayed a *negative* relationship between HRV and PPN rsFC with the amygdala. These contrasting correlations between HRV and arousal-related connectivity may help explain the heterogenous arousal-related symptom patterns characteristic of PTSD and PTSD + DS.

### HRV in PTSD and Its Dissociative Subtype

In the present study, we revealed reduced sympathetic-related HRV in individuals with PTSD + DS as compared to controls. Indeed, prior investigations have demonstrated a positive relationship between parasympathetic nervous system (PNS) activity and dissociation in response to threatening cues in other clinical populations, thus pointing to a close relationship between sympathoinhibition and dissociative symptomatology (Farina et al., 2015; Fitzpatrick and Kuo, 2015; Chou et al., 2018; Schäflein et al., 2018; Krause-Utz et al., 2019). Specifically, dissociation has been associated with parasympathetic overactivation, which has been thought to be linked to emotional detachment as expressed by symptoms of emotional numbing, depersonalization, and derealization (for an overview see Schauer and Elbert, 2015; Lanius et al., 2018, 2020; Terpou et al., 2019b).

By contrast, even though enhanced sympathetic responding has been related to hypervigilance (e.g., Pole, 2007), the present investigation revealed reduced SNS- and PNS-related HRV indices in individuals with PTSD as compared to controls. Here, it is important to note that arousal during resting state is influenced by both the SNS and the PNS (Shaffer and Ginsberg, 2017). Hence, the SNS-related HRV measure is not solely indicative of SNS activity but might also reflect PNS activity (Billman, 2011, 2013; Shaffer and Ginsberg, 2017). This may explain why we observed reduced HRV generally in individuals with PTSD as compared to controls (for meta-analyses see Nagpal et al., 2013; Chalmers et al., 2014; Sammito et al., 2015; Campbell et al., 2019; Schneider and Schwerdtfeger, 2020). Therefore, the present results should be interpreted with caution. Nevertheless, the present exploratory investigation emphasizes reduced ANS flexibility in individuals with PTSD and PTSD + DS, where reduced sympathetic activation was especially prominent in PTSD + DS.

### RsFC of the PPN and HRV in PTSD and Its Dissociative Subtype

The present investigation extends our previous findings on differential rsFC between the PPN and limbic and prefrontal regions in individuals with PTSD and PTSD + DS (Thome et al., 2019). Specifically, PPN rsFC with the amygdala and the parahippocampal gyrus were correlated differentially with HRV in PTSD + DS as compared to PTSD and controls, which may underlie the hyper- and more blunted arousal states commonly observed in PTSD and PTSD + DS, respectively (see also Wolf et al., 2012a,b; Seligowski et al., 2019 for review see Hansen et al., 2017; Fenster et al., 2018; Lanius et al., 2018; van Huijstee and Vermetten, 2018). The RAS is critical to generating arousal and innate reflexive responding, thereby ensuring adaptive behavior in threatening situations (Paus, 2000; Sarter et al., 2006; Maldonato, 2014; Venkatraman et al., 2017).

Given that the neural underpinnings of dissociation reside at least partly among evolutionarily conserved neurocircuitries, and that the phenomenon itself can be reliably observed across species during periods of overwhelming stress, it has become evident that dissociation as a construct may be adaptive, perhaps offering a way to escape psychologically so to avoid potential injury when fighting or fleeing are perceived as futile. In PTSD, dissociative responses are engaged often during trauma-related reexperiencing and are related to a reduced responsivity to environmental stimuli (Schauer and Elbert, 2015; Lanius et al., 2018; Seligowski et al., 2019; Terpou et al., 2019b). The latter may therefore restrict the opportunity to update prior learned stimulus-response contingencies (Ebner-Priemer et al., 2005; Krause-Utz et al., 2019). The correlational pattern of an enhanced synchronization of the RAS with the limbic system in individuals with PTSD + DS that are characterized by reduced autonomic flexibility could hence be interpreted as a neurophysiological substrate of restricted ability to adaptively interact with and hence update information related to the environment.

### Limitations

Several limitations of the current investigation are important to note. Firstly, brainstem structures are small and have gray and white matter distributions that are harder to delineate as compared to the cortex. Higher resolution fMRI scanning can improve the resolution of these structures. Although we did not use a high-resolution fMRI scanner, we implemented additional preprocessing measures to improve the resolution of the signal extracted from the PPN. It is also important to note that HRV measures were estimated based on pulse recordings, which sample RR intervals less sensitively than ECG recordings. While pulse data are a reliable marker of time and frequency features in healthy individuals, reliability was observed to be somewhat reduced in subjects with a cardiovascular disorder (Pinheiro et al., 2016). A re-examination with HRV measures based on ECG data could corroborate the present results.

## Conclusion

In the present exploratory study, we found that individuals with PTSD and PTSD + DS demonstrate reduced HRV as compared to controls, corroborating previous findings in the literature. Extending on this literature, we provide first evidence of a contrasting relationship between HRV and arousal-related brain connectivity in PTSD and PTSD + DS. Specifically, whereas a *positive* relationship between HRV and rsFC between the PPN and the amygdala was observed in participants with PTSD and controls, a *negative* relationship was observed between these same measures in PTSD + DS. This provides evidence of a contrasting pattern of arousal-related brain connectivity among participants with PTSD and PTSD + DS, offering a neurobiological lens to interpret hyper- and more blunted arousal states in PTSD and PTSD + DS, respectively. Treatments for PTSD will need to consider carefully these differential arousal states and their underlying neurocircuitry in order to manage arousal and emotion regulation among traumatized populations.

## Data Availability Statement

The datasets supporting the conclusions of this article will be made available by the authors upon request.

## Ethics Statement

The studies involving human participants were reviewed and approved by Western University, Canada. Written informed consent for participation was not required for this study in accordance with the national legislation and the institutional requirements.

## Author Contributions

RAL and JaT conceptualized the study and wrote the manuscript. JaT and MD performed the statistical analyses. BAT, JaT, MD, and RAL interpreted the data. BAT, JeT, MD, and MCM contributed to reading, revising, and approving the submitted manuscript. All authors contributed to the article and approved the submitted version.

## Conflict of Interest

The authors declare that the research was conducted in the absence of any commercial or financial relationships that could be construed as a potential conflict of interest.

## Publisher’s Note

All claims expressed in this article are solely those of the authors and do not necessarily represent those of their affiliated organizations, or those of the publisher, the editors and the reviewers. Any product that may be evaluated in this article, or claim that may be made by its manufacturer, is not guaranteed or endorsed by the publisher.

## Footnotes

1
http://www.fil.ion.ucl.ac.uk/spm

2
www.nitrc.org/projects/artifact_detect
