# Supplementary material for: Contrasting Associations Between Heart Rate Variability and Brainstem-Limbic Connectivity in Posttraumatic Stress Disorder and Its Dissociative Subtype: A Pilot Study
Source: Front Behav Neurosci. 2022 May 30;16:862192. doi: 10.3389/fnbeh.2022.862192 (PMC9190757; doi:10.3389/fnbeh.2022.862192)
Supplement: Supplementary file 1 [file Table_1.pdf]

Supplementary Material

Contrasting Associations between Heart Rate Variability and Brainstem-Limbic Connectivity in PTSD and its Dissociative Subtype: A Pilot Study

Running title: HRV and brainstem in PTSD

Janine Thome, PhD (janine.thome@zi-mannheim.de)[a,h,i], Maria Densmore, BSc (mdensmor@lawsonimaging.ca)[a,d], Braeden A. Terpou, PhD (terpouba@mcmaster.ca)[e,g], Jean Théberge, PhD (jtheberge@lawsonimaging.ca)[a,c,d], Margaret C. McKinnon, PhD (mckinno@mcmaster.ca)[e,f,g], Ruth A. Lanius, MD, PhD (ruth.lanius@lhsc.on.ca)[a,b,d,e,*]

[a] Department of Psychiatry, [b] Department of Neuroscience, [c] Department of Medical Biophysics, Western University, London, Ontario, Canada
[d] Imaging Division, Lawson Health Research Institute, London, Ontario, Canada
[e] Homewood Research Institute, Guelph, Ontario, Canada
[f] Mood Disorder Programs, St. Joseph's Healthcare, Hamilton, Ontario, Canada
[g] Department of Psychiatry and Behavioral Neurosciences, McMaster University, Hamilton, Ontario, Canada
[h] Department of Theoretical Neuroscience, [i] Clinic for Psychiatry and Psychotherapy, Central Institute of Mental Health Mannheim, Medical Faculty Mannheim, Heidelberg University, Germany

***Corresponding author:** Ruth Lanius, PhD, Department of Psychiatry, University of Western Ontario, Canada, Phone: 5196633306, Email: ruth.lanius@lhsc.on.ca

S1 Supplemental Methods

Methods: Clinical Measurements

Depressive symptomatology and childhood trauma severity were assessed with the Beck Depression Inventory (BDI; Beck, Guth, Steer, & Ball, 1997) and the childhood trauma questionnaire (CTQ; Bernstein et al., 2003), respectively. Trait dissociative experiences were assessed with the Multiscale Dissociation Inventory (MDI; Briere, Weathers, & Runtz, 2005). The extent of state anxiety (3 items of the State-Trait Anxiety Inventory; STAI; Spielberger, 2010) and state dissociation (Response to Script Driven Imagery Scale; RSDI; Hopper, Frewen, Van der Kolk, & Lanius, 2007) during the resting state scan were assessed immediately after the resting state session.

Methods: Exclusion criteria

Participants were excluded if they had implants or metal that do not comply with 3T fMRI safety standards for research, a history of head injury with a loss of consciousness, significant untreated medical illness, a history of neurological disorders, history of any pervasive developmental disorders, and pregnancy. PTSD individuals were further excluded if they reported a history of bipolar disorder, schizophrenia, or substance-use disorder six month prior to participation of the study.

Statistical analysis: Sample characteristics

Age, the severity of PTSD symptoms (CAPS total score), childhood traumatization (CTQ total score), trait dissociative experiences (MDI total score, MDI derealization subscale, MDI depersonalization subscale), state dissociative experiences (RSDI derealization and depersonalization), as well as state anxiety (STAI-S) was compared using one-way analysis of

variance (ANOVA; despite multivariate ANOVA has been applied to compare MDI, as well as CTQ subscales). Moreover, gender was contrasted using Kruskall-Wallis Test.

Statistical significance was set to $p<0.05$, and in case of significant effects, Bonferroni-corrected, post-hoc analyses were applied. All analyses were performed using SPSS (version 25; SPSS Inc., USA).

fMRI data preprocessing: brainstem and cerebellum

To enhance the signal extraction of the PPN via an improvement of the voxel-by-voxel normalization of the midbrain, lower brainstem, and cerebellum, functional and anatomical data were normalized to the spatially-unbiased infratentorial template (SUIT, version 3.1, Diedrichsen 2006; Diedrichsen et al., 2011) by applying the following steps: 1) whole-brain anatomical images were first segmented and then cropped, retaining only the cerebellum and brainstem; 2) The partial-brain anatomical images were normalized using the SUIT-normalize function that creates a nonlinear deformation map to the SUIT template by applying the cosine-basis approach introduced by Ashburner; 3) The realigned and resliced functional images (see 2.3.1) were normalized by applying the deformation matrix generated in step 2, cropped (retaining the cerebellum and brainstem only, i.e., functional partial-brain), and re-sliced to a voxel size of 1.5 x 1.5 x 1.5 mm^3; 4) partial-brain functional data were smoothed with a Gaussian filter of 4 mm full-width at half-maximum (FWHM) and band-pass filtered with a high-pass filter of .01 Hz and a low-pass filter of .08 Hz (Bär et al., 2016; Wagner et al., 2018).

S2 Results

Table S1. Within-Group Resting State Functional Connectivity of the Left Pedunculopontine Nuclei in Association with HF-HRV.

	L/R		Brain Region	k	Z	$p_{FWE\text{-}corr}$	p_{uncorr}	Peak MNI Coordinate x	y	z
**Controls**										
	L	↑	Anterior Cingulate Cortex	51	4.072	0.752	<.001	-2	30	18
	R	↑	Anterior Cingulate Cortex		3.454	1.000	<.001	2	24	26
	L	↑	Anterior Cingulate Cortex		3.201	1.000	0.001	-6	38	18
		↓	n.s.							
**PTSD**										
	L	↑	Superior Frontal Gyrus	20	4.188	0.570	<.001	-16	30	42
	L	↑	Heschl Gyrus	29	4.102	0.681	<.001	-38	-22	10
	L	↑	Postcentral Gyrus	13	3.779	0.965	<.001	-44	-22	36
	R	↑	Heschl Gyrus	29	3.758	0.972	<.001	42	-18	10
	R	↑	Cuneus	16	3.612	0.996	<.001	6	-86	16
	R	↑	Lingual Gyrus	11	3.521	0.999	<.001	16	-74	2

	↓	n.s.
**PTSD+DS**		
	↑	n.s.
	↓	n.s.

Whole-brain RsFC results are reported at a local significance threshold of $p<0.001$, uncorrected.

Legend: HF-HRV = high-frequency heart rate variability; PTSD = post-traumatic stress disorder; PTSD+DS = PTSD with the dissociative subtype; L = Left; R = Right; n.s.=no significant difference; k = cluster size; p_{uncorr} = p-value, uncorrected for multiple comparisons; $p_{FWEcorr}$ = p-value, corrected for multiple comparisons (family-wise error)

Table S2. Within-Group Resting State Functional Connectivity of the Right Pedunculopontine Nuclei in Association with HF-HRV.

L/R		Brain Region	k	Z	$p_{FWE-corr}$	p_{uncorr}	Peak MNI Coordinate x	y	z
**Controls**									
	↑	n.s.							
	↓	n.s.							
**PTSD**									
L	↑	Inferior Frontal Gyrus	44	4.643	0.139	<.001	-38	26	18
L	↑	Superior Temporal Gyrus	38	4.263	0.480	<.001	-48	-22	10
L	↑	Precentral Gyrus	131	4.188	0.577	<.001	-44	4	34
L	↑	Precentral Gyrus		3.919	0.887	<.001	-50	4	26
L	↑	Inferior Frontal Gyrus		3.569	0.998	<.001	-40	2	26
L	↑	Precentral Gyrus	10	3.851	0.934	<.001	-26	-4	48
R	↑	Amygdala	17	3.807	0.957	<.001	26	2	-22

L	↑	Inferior Frontal Gyrus	13	3.706	0.987	<.001	-34	32	0
R	↑	Middle Frontal Gyrus	17	3.493	1.000	<.001	32	44	26
R	↓	Superior Occipital Cortex	19	3.775	0.969	<.001	18	-84	18
R	↓	Middle Occipital Cortex		3.355	1.000	<.001	26	-86	16
R	↓	Precentral Gyrus	19	3.541	0.999	<.001	38	-24	50
**PTSD+DS**									
	↑		n.s.						
	↓		n.s.						

Whole-brain RsFC results are reported at a local significance threshold of $p<0.001$, uncorrected.

Legend: HF-HRV = high-frequency heart rate variability; PTSD = post-traumatic stress disorder; PTSD+DS = PTSD with the dissociative subtype; L = Left; R = Right; n.s.=no significant difference; k = cluster size; p_{uncorr} = p-value, uncorrected for multiple comparisons; $p_{FWEcorr}$ = p-value, corrected for multiple comparisons (family-wise error)

Table S3. Within-Group Resting State Functional Connectivity of the Left Pedunculopontine Nuclei in Association with LF-HRV.

L/R		Brain Region	k	Z	$p_{FWE-corr}$	p_{uncorr}	Peak MNI Coordinate x	y	z
**Controls**									
L	↑	Anterior Cingulate Cortex	13	3.740	0.984	0.000	-2	22	20
L	↑	Middle Frontal Gyrus	17	3.640	0.996	0.000	-38	48	26
R	↑	Middle Frontal Gyrus	12	3.557	0.999	0.000	52	42	14
	↓	n.s.							
**PTSD**									
L	↑	Orbitofrontal Gyrus	25	4.445	0.275	0.000	-32	38	-14
L	↑	Superior Frontal Gyrus	13	4.025	0.774	0.000	-18	18	42
R	↑	Superior Temporal Gyrus	10	3.870	0.918	0.000	44	-20	-2
R	↑	Posterior Cingulate Cortex	15	3.869	0.919	0.000	12	-44	24
L	↑	Superior Frontal Gyrus	10	3.859	0.925	0.000	-14	32	40
R	↑	Superior Frontal Gyrus	13	3.805	0.954	0.000	20	44	26
L	↑	Inferior Frontal Gyrus	22	3.795	0.959	0.000	-48	22	12
L	↑	Insula		3.305	1.000	0.000	-42	14	8

L	↑	Inferior Frontal Gyrus	10	3.756	0.973	0.000	-44	36	2
L	↑	Lingual Gyurs	10	3.725	0.981	0.000	-22	-74	-4
L	↑	Supplementory Motor Area	16	3.678	0.990	0.000	-4	-4	50
L	↑	Postcentral Gyrus	10	3.606	0.997	0.000	-50	-12	22
	↓		n.s.						
**PTSD+DS**									
	↑		n.s.						
	↓		n.s.						

Whole-brain RsFC results are reported at a local significance threshold of $p<0.001$, uncorrected.

Legend: LF-HRV = low-frequency heart rate variability; PTSD = post-traumatic stress disorder; PTSD+DS = PTSD with the dissociative subtype; L = Left; R = Right; n.s.=no significant difference; k = cluster size; p_{uncorr} = *p*-value, uncorrected for multiple comparisons; $p_{FWEcorr}$ = *p*-value, corrected for multiple comparisons (family-wise error)

Table S4. Within-Group Resting State Functional Connectivity of the Right Pedunculopontine Nuclei in Association with LF-HRV.

	L/R		Brain Region	k	Z	$p_{FWE-corr}$	p_{uncorr}	Peak MNI Coordinate x	y	z
**Controls**										
		↑	n.s.							
		↓	n.s.							
**PTSD**										
	R	↑	Heschl Gyrus	58	4.368	0.362	0.000	50	-18	8
	R	↑	Rolandic Operculum		3.358	1.000	0.000	54	-10	12
	R	↑	Insula	17	4.294	0.447	0.000	38	-20	2
	L	↑	Inferior Frontal Gyrus	25	4.271	0.475	0.000	-46	16	8
	R	↑	Middle Temporal Gyrus	51	4.237	0.519	0.000	50	-44	8
	R	↑	Middle Cingulate Cortex	25	4.075	0.728	0.000	10	16	36
	R	↑	Superior Parietal Cortex	28	4.002	0.813	0.000	22	-52	60
	R	↑	Superior Frontal Gyrus	17	3.982	0.833	0.000	24	6	52
	R	↑	Cerebellum Crus I	13	3.938	0.874	0.000	32	-64	-34
	R	↑	Cerebellum Crus I	41	3.900	0.905	0.000	30	-74	-26
	L	↑	Middle Frontal Gyrus	17	3.858	0.933	0.000	-34	40	16

L	↑	Inferior Frontal Gyrus		3.261	1.000	0.001	-40	34	10
L	↑	Superior Temporal Gyrus	74	3.849	0.938	0.000	-50	-20	8
L	↑	Superior Temporal Gyrus		3.743	0.980	0.000	-54	-28	10
L	↑	Superior Temporal Gyrus		3.605	0.997	0.000	-50	-16	0
L	↑	Supplementary Motor Area	15	3.836	0.945	0.000	-2	12	44
L	↑	Calcarine Sulcus	16	3.809	0.958	0.000	-18	-70	12
R	↑	Olfactory	26	3.727	0.983	0.000	26	8	-18
L	↑	Precentral Gyrus	19	3.722	0.984	0.000	-54	0	24
R	↑	Precuneus	11	3.701	0.988	0.000	4	-48	20
R	↑	Middle Frontal Gyrus	14	3.694	0.989	0.000	40	28	22
	↑	Cerebellum Vermis IV-V	10	3.692	0.990	0.000	0	-50	-8
L	↑	Caudate Nucleus	15	3.668	0.993	0.000	-20	-22	22
L	↑	Middle Frontal Gyrus		3.292	1.000	0.000	-22	20	48
L	↑	Inferior Frontal Gyrus	25	3.630	0.996	0.000	-54	30	16
R	↑	Inferior Frontal Gyrus		3.319	1.000	0.000	-48	36	16
R	↑	Caudate Nucleus	15	3.575	0.998	0.000	14	-8	18
R	↑	Inferior Frontal Gyrus	22	3.572	0.999	0.000	56	32	24
R	↑	Inferior Frontal Gyrus		3.160	1.000	0.001	50	28	18

	↑	Anterior Cingulate Cortex	28	3.564	0.999	0.000	0	38	18
L	↑	Insula	18	3.530	0.999	0.000	-36	6	0
L	↑	Inferior Frontal Gyrus	14	3.515	1.000	0.000	-38	2	22
L	↑	Superior Frontal Gyrus	14	3.493	1.000	0.000	-10	38	46
L	↑	Middle Frontal Gyrus	17	3.444	1.000	0.000	-30	48	26
L	↑	Angular Gyrus	11	3.281	1.000	0.001	-40	-62	24
	↓		n.s.						
**PTSD+DS**									
L	↑		16	3.597	0.937	0.000	-20	2	64
	↓		n.s.						

Whole-brain RsFC results are reported at a local significance threshold of $p<0.001$, uncorrected.

<u>Legend:</u> LF-HRV = low-frequency heart rate variability; PTSD = post-traumatic stress disorder; PTSD+DS = PTSD with the dissociative subtype; L = Left; R = Right; n.s.=no significant difference; k = cluster size; p_{uncorr} = p-value, uncorrected for multiple comparisons; $p_{FWEcorr}$ = p-value, corrected for multiple comparisons (family-wise error)

Table S5. Within-Group Resting State Functional Connectivity of the Left Pedunculopontine Nuclei in Association with RMSSD.

L/R		Brain Region	k	Z	$p_{FWE\text{-}corr}$	p_{uncorr}	Peak MNI Coordinate x	y	z
**Controls**									
L	↑	Anterior Cingulate Cortex	15	3.760	0.979	0.000	-2	22	20
L	↑	Anterior Cingulate Cortex		3.556	0.999	0.000	-4	30	18
R	↑	Anterior Cingulate Cortex	10	3.412	1.000	0.000	2	36	20
L	↑	Anterior Cingulate Cortex		3.217	1.000	0.001	6	28	24
	↓	n.s.							
**PTSD**									
	↑	n.s.							
L	↓	Anterior Cingulate Cortex		3.196	1.000	0.001	-12	30	-14
**PTSD+DS**									
	↑	n.s.							
	↓	n.s.							

Whole-brain RsFC results are reported at a local significance threshold of $p<0.001$, uncorrected.

Legend: RMSSD = root-mean square of successive differences; PTSD = post-traumatic stress disorder; PTSD+DS = PTSD with the dissociative subtype; L = Left; R = Right; n.s.=no significant difference; k = cluster size; p_{uncorr} = p-value, uncorrected for multiple comparisons; $p_{FWEcorr}$ = p-value, corrected for multiple comparisons (family-wise error)

Table S6. Within-Group Resting State Functional Connectivity of the Right Pedunculopontine Nuclei in Association with RMSSD.

L/R		Brain Region	k	Z	$p_{FWE\text{-}corr}$	p_{uncorr}	Peak MNI Coordinate x	y	z
**Controls**									
R	↑	Anterior Cingulate Cortex	10	3.601	0.998	0.000	8	22	20
	↓	n.s.							
**PTSD**									
L	↑	Inferior Frontal Gyrus	71	4.559	0.190	0.000	-38	28	18
L	↑	Middle Occipital Gyrus	13	3.939	0.871	0.000	-28	-80	12
L	↑	Inferior Frontal Gyrus	66	3.706	0.987	0.000	-48	14	28
L	↑	Precentral Gyrus		3.506	1.000	0.000	-48	2	28
L	↑	Precentral Gyrus		3.410	1.000	0.000	-42	4	34
L	↑	Inferior Frontal Gyrus	16	3.666	0.992	0.000	-34	32	2
L	↑	Superior Temporal Gyrus	18	3.664	0.993	0.000	-48	-22	12

	↓	n.s.
**PTSD+DS**		
	↑	n.s.
	↓	n.s.

Whole-brain RsFC results are reported at a local significance threshold of $p<0.001$, uncorrected.

Legend: RMSSD = root-mean square of successive differences; PTSD = post-traumatic stress disorder; PTSD+DS = PTSD with the dissociative subtype; L = Left; R = Right; n.s.=no significant difference; k = cluster size; p_{uncorr} = p-value, uncorrected for multiple comparisons; $p_{FWEcorr}$ = p-value, corrected for multiple comparisons (family-wise error)

S3 References

Bär KJ, de la Cruz F, Schumann A, Koehler S, Sauer H, Critchley H, et al. Functional connectivity and network analysis of midbrain and brainstem nuclei. Neuroimage. 2016;134:53-63.

Beck, A. T., Guth, D., Steer, R. A., & Ball, R. (1997). Screening for major depression disorders in medical inpatients with the Beck Depression Inventory for Primary Care. *Behav Res Ther, 35*(8), 785-791

Bernstein, D. P., Stein, J. A., Newcomb, M. D., Walker, E., Pogge, D., Ahluvalia, T., . . . Desmond, D. (2003). Development and validation of a brief screening version of the Childhood Trauma Questionnaire. *Child Abuse Negl, 27*(2), 169-190.

Briere, J., Weathers, F. W., & Runtz, M. (2005). Is dissociation a multidimensional construct? Data from the Multiscale Dissociation Inventory. *Journal of Traumatic Stress: Official Publication of The International Society for Traumatic Stress Studies, 18*(3), 221-231.

Diedrichsen J. A spatially unbiased atlas template of the human cerebellum. NeuroImage. 2006;33(1):127-38.

Diedrichsen J, Maderwald S, Küper M, Thürling M, Rabe K, Gizewski E, et al. Imaging the deep cerebellar nuclei: a probabilistic atlas and normalization procedure. NeuroImage. 2011;54(3):1786-94.

Hopper, J. W., Frewen, P. A., Van der Kolk, B. A., & Lanius, R. A. (2007). Neural correlates of reexperiencing, avoidance, and dissociation in PTSD: Symptom dimensions and emotion dysregulation in responses to script-driven trauma imagery. *Journal of traumatic stress, 20*(5), 713-725.

Wagner G, Krause-Utz A, de la Cruz F, Schumann A, Schmahl C, Bar KJ. Resting-state functional connectivity of neurotransmitter producing sites in female patients with borderline personality disorder. Progress in neuro-psychopharmacology & biological psychiatry. 2018;83:118-26.

Spielberger, C. D. (2010). State-Trait anxiety inventory. *The Corsini encyclopedia of psychology*, 1-1.
